# The diagnostic and predictive potential of personality traits and coping styles in major depressive disorder

**DOI:** 10.1186/s12888-022-03942-y

**Published:** 2022-04-28

**Authors:** Cyrus S. H. Ho, J. Chua, Gabrielle W. N. Tay

**Affiliations:** 1 Department of Psychological Medicine, National University Health System, Singapore, Singapore; 2 Department of Psychological Medicine, Yong Loo Lin School of Medicine, National University of Singapore, Singapore, Singapore; 3 Department of Psychology, Faculty of Arts and Social Sciences, National University of Singapore, Singapore, Singapore

**Keywords:** Major depressive disorder, Diagnostic, Prediction, Personality traits, Coping, Psychological testing

## Abstract

**Background:**

Major depressive disorder (MDD) is a global public health concern that is notably underdiagnosed and undertreated due to its complexity and subjective diagnostic methods. A holistic diagnostic procedure, which sufficiently considers all possible contributors to MDD symptoms, would improve MDD diagnosis and treatment. This study aims to explore whether personality and coping styles can predict MDD status and differentiate between depressed patients and healthy individuals.

**Methods:**

Seventy healthy controls (*N* = 54 females) were matched to 70 MDD patients for age, sex, ethnicity, and years of education. MDD severity was measured using the Hamilton Depression Rating Scale, while personality traits and coping styles were measured by the Ten-Item Personality (TIPI) and Brief COPE questionnaires, respectively. Logistic regression analyses were conducted to investigate the diagnostic and predictive potential of personality and coping styles. Receiver operating characteristic (ROC) analyses were also conducted to examine their discriminative ability to distinguish between depressed and healthy individuals.

**Results:**

Introversion, lack of organisation skills, and neuroticism were statistically significant in predicting MDD status. Dysfunctional coping strategies, such as denial and self-blame, were also shown to significantly predict MDD status. ROC analyses found both the TIPI questionnaire (AUC = 0.90), and dysfunctional coping (as measured by Brief COPE) (AUC = 0.90) to be excellent predictors of MDD.

**Conclusions:**

Our findings demonstrate the diagnostic and predictive potential of personality and coping styles for MDD in the clinical setting. They also demonstrate the remarkable ability of personality and coping styles to differentiate between depressed patients and healthy controls.

**Supplementary Information:**

The online version contains supplementary material available at 10.1186/s12888-022-03942-y.

## Background

Major depressive disorder (MDD) is currently the most common and pervasive psychiatric disorder in most societies worldwide, affecting over 300 million people of all ages and nationalities [1, 2]. MDD is a multifaceted condition characterised by a wide range of psychobiological symptoms, such as prolonged sadness, anhedonia, sleep and appetite disturbances, fatigue or loss of energy, excessive feelings of guilt or worthlessness and memory problems; these significantly affect one’s ability to function [3, 4]. Severe cases of MDD may also present with psychotic symptoms and are a risk factor for suicide [5, 6]. Given the wide range of symptoms, patients with MDD vary considerably in clinical presentation and treatment responses [7]. At present, MDD is one of the principal causes of disability globally, and it is projected to be the leading contributor to disease burden worldwide by 2030 [2, 8]. Its underlying etiology and pathophysiology, however, remain relatively poorly understood due to its complex nature, thus resulting in notable underdiagnosis and undertreatment [9–11]. It is therefore important to look into ways to improve the diagnosis and treatment of MDD.

At present, there is no clearly established consensus for how MDD should be diagnosed in the clinical setting [10]. Clinicians usually diagnose MDD in patients as guided by the Fifth Edition of the *Diagnostic and Statistical Manual of Mental Disorders* (DSM-5) [12] and their professional clinical expertise, in conjunction with patients’ responses to clinical-depression-related questionnaires, such as the Patient Health Questionnaire 9 [13]. Some patients, however, may not be forthcoming about their symptoms, especially suicidal ideation [14, 15]. Given the subjective nature of these methods of assessment, the accuracy of MDD diagnoses cannot be ascertained. Heterogeneity between patients with MDD, despite its clinical relevance, also cannot be captured accurately [16]. Furthermore, only 50% of patients with MDD respond adequately to treatment, and an even lower percentage of 33% attain remission, despite being treated with optimal medication based on measurement-based care and consensus guidelines [17, 18]. To improve MDD diagnoses and treatment, it is important to employ a more holistic and empirical approach when conducting clinical interviews with patients, such that all possible contributors to MDD, such as personality types and coping styles, are sufficiently explored in making a diagnosis.

Personality is one such factor that contributes to MDD and can be defined as biological, early-emerging, and individual differences in emotions and their regulation that develop and change over the lifespan in response to maturation and circumstances [19]. It is considered to be a significant determinant of psychological wellbeing [20, 21]. Personality traits are most commonly classified under the ‘Big Five’ personality dimensions typically used to measure individual differences in personality: extraversion (versus introversion), which reflects talkativeness, assertiveness, and activity level; agreeableness (versus antagonism), which assesses cooperativeness and compassion; conscientiousness (versus lack of organisation skills), which encompasses order, goal orientation, and discipline; neuroticism (versus emotional stability), which denotes negative affect; and openness to experience (versus close-mindedness), which measures intellectual curiosity and creativity [22, 23]. Some personality traits predispose individuals to developing psychiatric disorders, while others increase the likelihood of treatment resistance [19, 24, 25]. They can also be informative markers of risk for MDD [26]. Neuroticism, for example, has been noted to associate positively with personality disorders and found to be a vulnerability factor for comorbid psychiatric disorders [27–29]. For MDD specifically, neuroticism is a well-established risk factor in the DSM-5, given that individuals with high neuroticism tend to display emotional instability, higher reactivity to stress, and proneness to anxiety, all of which are symptoms associated with MDD [30–32]. There is also evidence that neuroticism accounts for cognitive traits such as rumination and evaluation, which have a negative impact on psychopathology [33]. On the other hand, extraversion, as characterised by sociability, assertiveness, and high energy levels, has demonstrated an inverse relationship with MDD [34, 35]. In other words, a lower incidence of MDD was found amongst individuals scoring higher on extraversion. Much less is known about how MDD is associated with the other personality dimensions [29, 36].

Coping is another factor that contributes to MDD and has been defined as a process in which cognitive and behavioural efforts are made to manage specific internal and/or external sources of psychological stress [37]. Coping strategies can be broadly classified into three styles (or categories), as defined by Lazarus and Folkman (1984) and Suls and Fletcher (1985): problem-focused coping, which employs efforts to change stressful circumstances caused by individual–environment interactions; emotion-focused coping, which includes actions and thoughts aimed at lessening the emotional consequences of stress; and dysfunctional coping, which encompasses behaviours and cognitions intended to divert attention and stress from its source [38–41]. These strategies can also be categorised based on whether they are adaptive or maladaptive [42]. Coping strategies have been shown to be potentially important moderators and mediators in the contribution of psychosocial stress to MDD and suicidal ideation, and vice versa [43]. Coping styles may also influence the persistence of psychotic experiences and the possible development of clinical psychotic disorders [44]. The use of emotion-focused coping, in which strategies focus on altering experiences of negative emotions resulting from a stressful source, has been linked to greater depressive symptom severity [37, 45]. Individuals who used dysfunctional coping strategies more often were also at a higher risk of being diagnosed with MDD [46].

Research findings demonstrate that how an individual copes with the problems they encounter may be influenced by personality traits [47], and associations between specific personality traits and coping have also been established in the literature. A study by Uehara and colleagues, for example, found a positive association between emotion-focused coping and neuroticism; it also found a positive association between problem-focused coping and extraversion [48]. Several other studies investigating the association between the Big Five personality traits and coping styles also found that extraverted individuals had a higher tendency to employ problem-focused coping strategies, whilst individuals who scored higher on the neuroticism scale were more likely to employ emotion-focused coping strategies, which could be explained by their higher reactivity to stress and the intensity of their experiences of negative emotions [47, 49, 50]. Given these associations, it would be important to consider both personality traits and coping styles in the diagnostic process for MDD in the clinical setting.

To the best of our knowledge, there is a dearth of studies pertaining to personality and coping styles and how they are associated with and/or can predict psychiatric disorders, specifically MDD, in both the Singaporean and wider non-Western contexts [51, 52]. As most of such studies have been conducted amongst Western populations, it is unclear if their findings can be generalised to populations of other nationalities and cultures. Furthermore, questionnaires regarding personality and coping styles have not yet been explored as tools to differentiate depressed patients from healthy controls (HCs). Our study therefore aims to fill this gap by generating fresh insight into whether personality, specifically the Big Five personality dimensions, and coping styles, are able to, firstly, predict MDD status and, secondly, differentiate between depressed patients and HCs, given that both personality and coping have shown to be contributing factors to MDD. We will do this by comparing a sample of patients recruited from the outpatient psychiatry clinic of the tertiary university hospital in Singapore to healthy individuals. The associations between the different personality traits and coping strategies are also explored in our study, as personality and coping styles have been found, in several instances, to play interactive roles in influencing psychological health outcomes measured by both clinical symptoms and subjective wellbeing [53], yet previous MDD-focused studies have mainly only explored one or the other. Taken together, the findings from our study would be crucial for the improvement of MDD diagnoses and treatment, as we expect personality and coping styles to have implications on the disease progression and treatment response of depressed patients [19, 53].

## Materials and methods

### Participants

A total of 70 patients with MDD (*n* = 54 females) and 70 HCs (healthy controls; *n* = 54 females), all of whom were English-speaking and aged between 21 and 50 years, were included in this study. All patients were recruited from the outpatient psychiatry clinics at a university hospital in Singapore, where they had been diagnosed with MDD by a psychiatrist in accordance with the criteria in the DSM-5 [12]. HCs were recruited from the community and, after being matched with patients for sex, age (±7), ethnicity, and years of education, underwent the same study procedures. Individuals were excluded if they had conditions that could affect the central nervous system, including cerebrovascular diseases, respiratory diseases, hepatic diseases, kidney diseases, cancer, epilepsy, or intellectual disability, as well as a history of psychiatric and/or neurological disorders.

Written questionnaires were completed during individual study visits. Study details were fully explained to participants, and their written, informed consent was obtained. All procedures contributing to this work comply with the ethical standards of the Declaration of Helsinki and the ethical principles in the Belmont Report. Approval was granted by the Domain Specific Review Board of the National Healthcare Group, Singapore (protocol number 2019/00141). All questionnaires collected were de-identified.

### Measures

#### Demographic and clinical information

Sociodemographic information was collected from all participants. HCs were also asked to indicate if they had family members with a history of psychiatric disorders. For patients with MDD, their clinical information (both medical and psychiatric history) was obtained and synthesised from both computerised and physical records.

#### Hamilton rating scale for depression (HAM-D)

The HAM-D is a 21-item clinician-administered questionnaire designed to measure the severity of symptoms in patients diagnosed with MDD: depressed mood; guilt; suicidality; early, middle, and late insomnia; anhedonia; psychomotor retardation; agitation; psychological and somatic anxiety; gastrointestinal and general somatic symptoms; genital symptoms; hypochondriasis; insight into condition; weight loss; diurnal variation; derealisation; paranoid symptoms and obsessional and compulsive symptoms [54]. Every item is scored on a 4-point scale, based on the assessment and judgment of the clinician derived from the information elicited from the patient, including nonverbal cues. According to Hamilton (1960), scores of the last four items are generally excluded from the calculation of the total HAM-D score (HAM-D 17) due to the rarity of their occurrences; diurnal variation also measures depression type rather than severity.

The HAM-D 17 score is used to determine depression severity: mild (8-16), moderate (17-23), and severe (> = 24) [55]. The first 17 items of the HAM-D can also be categorised into five subscales: insomnia (items measuring early, middle, and late insomnia), anxiety (items measuring psychological and physiological anxiety), somatic (items measuring gastrointestinal and general somatic symptoms), melancholia (items measuring depressed mood, guilt, anhedonia, psychomotor retardation, psychological anxiety, and general somatic symptoms), and response-based (items measuring depressed mood, guilt, suicidality, anhedonia, psychomotor retardation, psychological anxiety, and general somatic symptoms). In the present study, this questionnaire presented excellent internal reliability with a Cronbach’s alpha value of 0.94.

#### Ten-item personality inventory (TIPI)

The Ten-Item Personality Inventory is a validated 10-item self-administered questionnaire designed to measure personality in relation to the ‘Big Five’ personality dimensions—extraversion, agreeableness, conscientiousness, neuroticism, and openness to experience [56]. Items capture a personality dimension and are scored on a 7-point Likert scale with “1” representing “Disagree strongly” and “7” representing “Agree strongly”. The overall score of each Big Five personality dimension subscale is the sum of scores of two items, one of which is reverse-coded. In the present study, this questionnaire presented good internal reliability with a Cronbach’s alpha value of 0.74.

#### Brief coping orientation to problems experienced (COPE)

The Brief COPE is a validated 28-item self-administered questionnaire designed to measure the use of effective and ineffective ways to cope with a stressful life event, which are classified into a total of 14 theoretically or empirically grounded coping strategies (i.e., 2 items per strategy): active coping, planning, positive reframing, acceptance, humour, religion, use of emotional support, use of instrumental support, self-distraction, denial, venting, substance use, behavioural disengagement, and self-blame [57]. Every item is a statement that is scored on a 4-point Likert scale, with “1” representing “Very seldom” and “4” representing “Very often”. The overall score of each coping strategy is the sum of the scores of two different items.

Coping strategies are subsequently categorised into three coping styles—problem-focused coping (active coping, use of instrumental support, and planning), emotion-focused coping (use of emotional support, positive reframing, humour, acceptance, and religion), and dysfunctional coping (self-distraction, denial, substance use, behavioural disengagement, venting, and self-blame)—and the overall score for each subscale is the sum of scores of its constituent coping strategies. These coping strategies are also classified as being maladaptive (self-distraction, denial, substance use, behavioural disengagement, venting, and self-blame) or adaptive (active coping, use of instrumental support, use of emotional support, positive reframing, planning, humour, acceptance, and religion). A maladaptive–adaptive score is also calculated from the difference between the average maladaptive and adaptive scores. In the present study, this questionnaire presented good internal reliability with a Cronbach’s alpha value of 0.79.

### Statistical analyses

All statistical tests were two-tailed, with a significance level of *p* < 0.05. Analyses were conducted using R version 4.0.2 [58]. Comparisons of demographic and clinical characteristics of interest were conducted using independent-sample *t* tests for continuous variables and chi-square tests of independence for categorical variables. Associations between HAM-D total score with each of the TIPI personality dimensions and categories of coping strategies as defined by Brief COPE were explored using bivariate Pearson’s correlations. To correct for multiple comparisons, the Holm–Bonferroni method was used. Additionally, multiple linear regression analyses were conducted to explore the predictive capacities of each of the personality dimensions and categories of coping strategies on the HAM-D total score.

Multiple logistic regression analyses were conducted to investigate whether certain coping strategies or personality types could be useful in predicting MDD status. Odds ratios (ORs) with 95% confidence intervals (CI) were calculated to explore the differences between the patient and HC groups using specific dimensions of personality (extraversion, conscientiousness, and emotional stability) and coping strategies (denial, substance abuse, venting, positive reframing, self-blame), with MDD status as the dependent variable. Receiver operating characteristic (ROC) analyses were performed to examine the accuracy of either personality or coping styles in differentiating depressed patients from HCs. Sensitivities and specificities were determined using the optimum cut-off point as defined by the point on the ROC curve closest to (0, 1).

## Results

### Sample characteristics

HCs and depressed patients did not differ in age, sex, or ethnicity (*p* > .05; Table 1).

**Table 1:** Demographic and Clinical Characteristics

	HC (*n* = 70)	MDD (*n* = 70)	*p* value
Age (years)	28.2 (SD 7.3)	28.3 (SD 7.2)	.926
Sex			1.000
Male	16 (22.9%)	16 (22.9%)	
Female	54 (77.1%)	54 (77.1%)	
Ethnicity			1.000
Chinese	45 (64.3%)	45 (64.3%)	
Malay	15 (21.4%)	15 (21.4%)	
Indian	9 (12.9%)	9 (12.9%)	
Eurasian	1 (1.4%)	1 (1.4%)	
Education (years)	15.6 (SD 1.2)	14.5 (SD 1.8)	**< .001**
HAM-D	1.9 (SD 2.5)	19.8 (SD 5.4)	**< .001**
Mild (8 – 16)	4 (5.7%)	20 (28.6%)	
Moderate (17 – 23)	0	30 (42.9%)	
Severe (≥ 24)	0	19 (27.1%)	
Family psychiatric history	17 (24.3%)	30 (42.9%)	**.032**
Age at onset (years)		20.7 (SD 7.5)	
Duration of illness (years)		7.9 (SD 6.5)	
Past admission to psychiatric ward		16 (22.9%)	
Past suicide attempt		32 (45.7%)	
Pharmacotherapy		60 (85.7%)	
Antidepressants		60 (100%)	
Anxiolytics and sedatives		11 (18.3%)	
Antipsychotics		11 (18.3%)	
Mood stabiliser		4 (6.7%)	
Fluoxetine equivalent dose (mg/day)		72.6 (SD 71.8)	
Diazepam equivalent dose (mg/day)		38.6 (SD 74.3)	
Chlorpromazine equivalent dose (mg/day)		40.1 (SD 29.9)	

*p*-values ≤ .05 are in bold

However, there were significant differences in years of education, family psychiatric history, and HAM-D scores. Depressed patients had fewer years of education than HCs (*t* = 3.94, *p* < .001). A greater proportion of depressed patients had family psychiatric history compared to HCs (*χ* ^2^ (1, *N* = 140) = 4.61, *p* = .032). As expected, depressed patients had significantly elevated HAM-D scores compared to HCs (*t* = 25.3, *p* < .001). Among depressed patients, 77.1% had no history of admission into a psychiatric ward. In total, 85.7% of patients were on pharmacotherapy (*n* = 60), all of whom were on antidepressants; some were also on antipsychotics, anxiolytics, and sedatives or mood stabilisers (see Table 1).

HCs and depressed patients differed significantly in personality and coping. For personality, depressed patients showed greater levels of introversion, antagonism, disorganisation, neuroticism, and close-mindedness compared to HCs. For coping, depressed patients exhibited lower levels of problem-based and emotion-based coping, and elevated levels of dysfunctional coping (see Table 2).

**Table 2:** Mean (SD) of TIPI and Brief COPE scores across groups

	HC (SD)	MDD (SD)	***p-***value
**TIPI**			
**Extraversion**	4.34 (1.56)	3.06 (1.53)	< .001
**Agreeableness**	5.36 (1.04)	4.41 (1.36)	< .001
**Conscientiousness**	5.34 (1.10)	4.09 (1.66)	< .001
**Emotional stability**	4.80 (1.25)	2.41 (1.11)	< .001
**Open mindedness**	4.99 (1.05)	4.34 (1.46)	.003
**Brief COPE**			
**Problem-based coping**	16.71 (3.59)	15.24 (4.09)	.025
**Emotion-based coping**	25.80 (5.37)	23.06 (4.55)	.001
**Dysfunctional coping**	20.09 (4.05)	28.06 (4.70)	< .001

### Personality traits (TIPI)

#### Associations with HAM-D Total score

HAM-D total score was negatively associated with extraversion (*r(138)* = − 0.33), agreeableness (*r(138)* = − 0.37), conscientiousness *(r(138)* = − 0.39), emotional stability (*r(138)* = − 0.70), and open-mindedness (*r(138)* = − 0.24). All *p*-values = < 0.001. (Supplementary Fig. 1).

Multiple regression analyses with HAM-D total score as the dependent variable revealed that extraversion, β = − 0.14, *t*(134) = − 2.17, *p* = .032, conscientiousness, β = − 0.17, *t*(134) = − 2.66, *p* = .009, and emotional stability, β = − 0.57, *t*(134) = − 7.96, *p* < .001, significantly inversely predicted HAM-D total score (Supplementary Table 1). The total explained variance was 54.4% (*F*(5, 134) = 31.97, *p* < .001).

#### Logistic regression analysis

Introversion (β = 0.72, *p* = 0.013), lack of organisation skills (β = 0.64, *p* = 0.039), and neuroticism (β = 2.18, *p* < 0.001) were found to be statistically significant in predicting MDD status (Table 3).

**Table 3:** Logistic regression analyses of MDD status on TIPI personality types

	Standardised β	*p*-value	Odds Ratio	95% CI
Introversion	0.72	.013	2.06	[1.19, 3.78]
Lack of organization	0.64	.039	1.90	[1.06, 3.59]
Neuroticism	2.18	< .001	8.82	[4.35, 21.34]
Constant	- 0.01	.962		

Odds of being depressed increased 2.06 times for every SD increase in introversion, 1.90 times for every SD increase in disorganisation, and 8.82 times for every SD increase in neuroticism; amongst the Big Five personality traits, odds of being depressed increased most markedly following an SD increase in neuroticism.

#### Differentiating MDD patients from HCs using TIPI

**Fig. 1:**
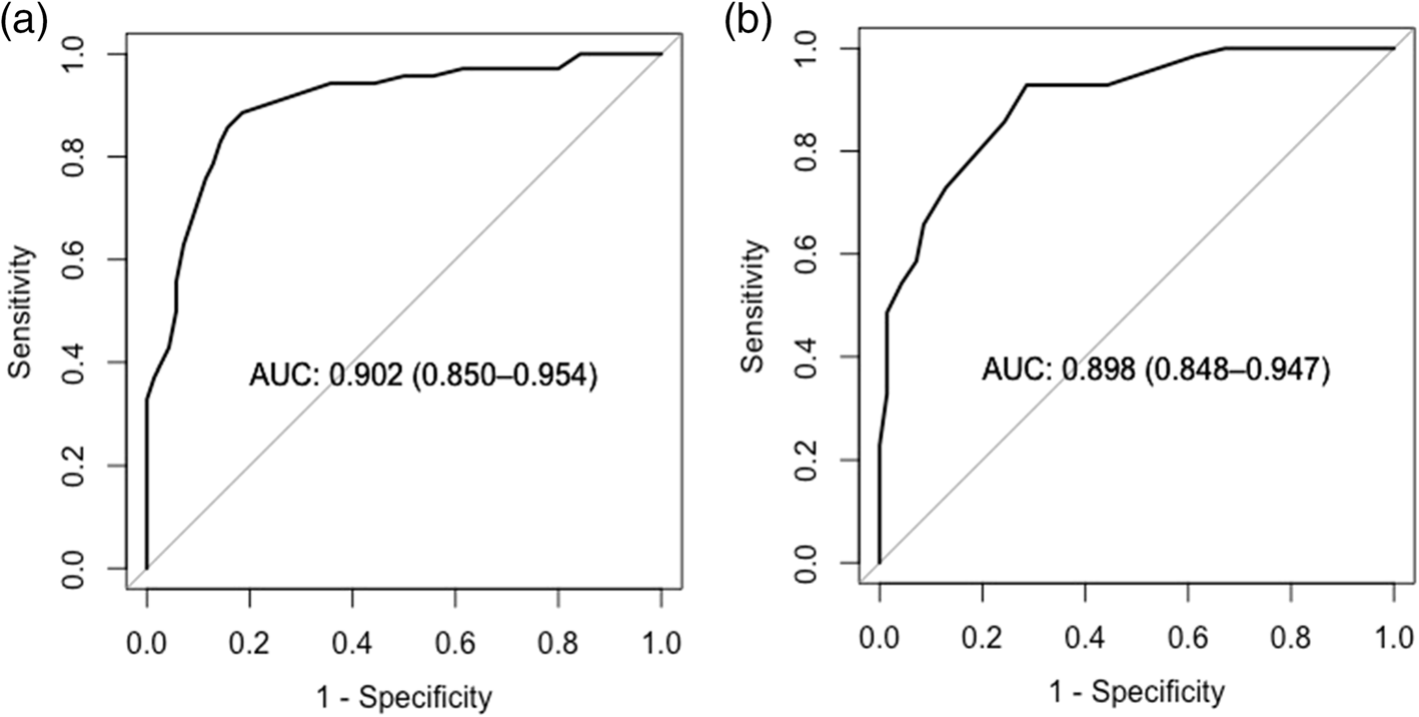
Receiver Operating Characteristic (ROC) analysis of the (**a**) TIPI, and (**b**) dysfunctional coping, scores between patients with MDD and HCs

The area under the curve (AUC) was 0.90 [95% CI = (0.85, 0.95)], indicating that the TIPI questionnaire was able to distinguish between depressed patients and HCs with excellent accuracy (see Fig. 1a).

Using a threshold value of 22.5 and below predicting for patients with MDD, TIPI correctly classified 85.7% of patients (proportion of patients/measurements: 60/70) and 84.3% of HCs (proportion of controls/measurements: 59/70; positive predictive value (PPV) = 0.85; negative predictive value (NPV) = 0.86).

### Coping strategies (brief COPE)

#### Associations with HAM-D Total score

HAM-D total score was negatively associated with problem-based (*r* = − 0.19, *p* = .020), and emotion-based coping (*r(138)* = − 0.28, *p* < .001) but was also strongly positively associated with dysfunctional (*r(138)* = 0.69, *p* < .001) and maladaptive–adaptive coping (*r(138)* = 0.73, *p* < .001; see Supplementary Fig. 3).

Multiple regression analyses with HAM-D total score as the dependent variable revealed that emotion-based coping significantly inversely predicted HAM-D total score, β = − 0.29, *t*(134) = − 4.50, *p* < .001, while dysfunctional coping significantly predicted HAM-D total score, β = 0.71, *t*(134) = 12.94, *p* < .001 (see Supplementary Table 2). The total explained variance was 58.8% (*F*(3, 136) = 64.62, *p* < .001).

#### Logistic regression analysis

Self-distraction (β = 0.85, *p* = 0.050), denial (β = 2.06, *p* = 0.006), substance use (β = 1.49, *p* = 0.023), venting (β = 1.54, *p* = 0.015), positive reframing (β = 2.23, *p* < 0.001), and self-blame (β = 1.34, *p* = 0.011) were found to be statistically significant in predicting MDD status (Table 4).

**Table 4:** Logistic regression analyses of MDD status on Brief COPE coping styles

	Standardised β	*p*-value	Odds Ratio	95% CI
Self-distraction	0.85	.050	2.35	[1.05, 6.03]
Denial	2.06	.006	7.82	[2.12, 41.19]
Substance use	1.49	.023	4.43	[1.53, 20.36]
Venting	1.54	.015	4.67	[1.52, 19.33]
Negative thinking	2.23	< .001	9.32	[2.96, 43.91]
Self-blame	1.34	.011	3.81	[1.44, 11.94]
Constant	0.82	.066		

Odds of being depressed increased 2.35 times for every unit increase in self-distraction, 7.82 times for every unit increase in denial, 4.43 times for every unit increase in substance use, 4.67 times for every unit increase in venting, 9.32 times for every unit increase in negative thinking, and 3.81 times for every unit increase in self-blame. Engaging in negative thinking (as opposed to positive reframing) led to the greatest increase in odds of being depressed.

#### Differentiating MDD patients from HCs using brief COPE

The AUC was 0.90 [95% CI = (0.85, 0.95)], indicating that dysfunctional coping was able to distinguish between depressed patients and HCs with excellent accuracy (see Fig. 1b). Using a threshold value of 24 and above predicting for patients with MDD, dysfunctional coping correctly classified 85.7% of patients (proportion of patients/measurements: 60/70) and 75.7% of HCs (proportion of controls/measurements: 53/70; PPV = 0.78; NPV = 0.84).

## Discussion

Our study aimed to explore whether personality traits and coping styles can predict MDD status and/or differentiate between depressed patients and healthy controls. To the best of our knowledge, this is the first study to do this in the Singaporean context. Our findings will therefore be able to provide more comprehensive evidence of the potential of personality traits and coping styles, as measured by the TIPI and Brief COPE questionnaires, to diagnose MDD in the clinical setting or distinguish between depressed patients and healthy individuals. Of the personality dimensions and coping styles that demonstrated statistically significant associations with the HAM-D total score and HAM-D categories, some were found to predict MDD status in a statistically significant manner. Furthermore, both the aforementioned questionnaires were able to distinguish between depressed patients and HCs with considerable accuracy.

### Personality traits and MDD

The mean scores for each of the Big Five personality dimension subscales derived from our sample are in line with the normative scores of an Asian subsample consisting of 333 undergraduate students from the University of Texas [56]. We also found that MDD patients generally scored lower on all subscales, particularly emotional stability. This is in agreement with the existing literature, where depressed patients repeatedly demonstrated significantly lower levels of extraversion, openness to experience, and/or conscientiousness, along with higher levels of neuroticism, when compared to healthy individuals [59–61].

It is possible that depressed patients generally experience higher levels of neuroticism, as they are less capable of regulating the negative emotions that they experience [62]. Thus, their lower levels of extraversion, openness to experience, and conscientiousness could be a result of them constantly experiencing negative emotions, such as sadness, hopelessness, and anhedonia, which are core depressive symptoms [63]. Furthermore, depressed patients could demonstrate lower levels of agreeableness because they are less likely to engage in prosocial behaviours, which are often motivated by positive emotions [64, 65]. They could also be more cynical and sceptical, hence maintaining a cautious approach when interacting with or considering whether to extend help to others [66].

Our study also found that being more neurotic, less conscientious, and more introverted are personality traits that can significantly predict positive MDD status. These findings are consistent, first of all, with extensive research that has found higher levels of neuroticism strongly predicting MDD status [67, 68]. This can be explained by the fact that neurotic individuals typically experience more negative affect (i.e., are more anxious and insecure, and also more reactive to stressors) and are therefore more vulnerable to adverse life experiences [69, 70]. They also often experience the lasting effects of these negative emotions, which they have the tendency to cope with using maladaptive emotion regulation strategies, hence placing them at higher risk of MDD [62].

Our findings are also in agreement with previous studies highlighting how conscientiousness negatively predicts depressive symptoms in HCs and depressed patients alike [26, 66]. Smith, Barstead, and Rubin (2017) posit that conscientiousness is often accompanied by strong self-regulation and the capacity for effortful control of attention and behaviour and can thus protect against experiences of negative emotions. As such, lower conscientiousness could predict MDD due to more frequent (and sustained) experiences of negative emotions being a common symptom of MDD [71]. It would also be useful, however, to consider the view of Klein, Kotov, and Bufferd (2011) that lower levels of conscientiousness may not predict MDD status directly but rather increase individuals’ exposure to stress and negative life events, which could then culminate in MDD. Finally, our finding that lower levels of extraversion predict MDD also confirms the association established in several earlier studies [34, 35, 72]: more introverted individuals are less assertive, more socially withdrawn, and less likely to experience positive emotions, all of which have previously demonstrated associations with MDD [19, 73].

The predictive potential of individual personality traits for a positive MDD status as highlighted by this study should be interpreted with caution, as earlier studies suggest certain synergistic relationships amongst them (i.e., they form specific personality profiles), or between them and other biological or environmental factors [36, 74]. Taken together, our findings broadly support the work of Boudouda and Gana (2020), who found a significant three-way interaction between neuroticism, conscientiousness, and extraversion, where high levels of conscientiousness and extraversion were protective against the deleterious effects of high levels of neuroticism on depressive mood.

The results of our ROC analyses also revealed that the TIPI questionnaire is able to distinguish between depressed patients and HCs with excellent accuracy. A threshold value of 22.5 had optimal sensitivity and specificity values for screening for, and classifying, depressed patients.

### Coping styles and MDD

A comparison between depressed patients and HCs on the mean scores of each of the three coping style subscales found that depressed patients generally scored lower on the problem-based coping and emotion-based coping subscales, but significantly higher on the dysfunctional coping subscale. The elevated dysfunctional coping subscale scores are largely consistent with the literature, where a positive association between MDD and dysfunctional coping is well established [75]. Depressed patients were commonly found to engage in behavioural disengagement, self-blame, and/or denial, all of which are dysfunctional coping strategies [76–79]. Earlier studies conducted amongst women diagnosed with perinatal and postpartum depression (PPD) also found a positive association between PPD and dysfunctional coping [80–82]. This association may be explained by how dysfunctional coping strategies, despite their tendency to maintain or strengthen MDD, are able to reduce the experience of its symptoms in the short term, hence providing depressed patients with the relief they desire, albeit temporarily [83]. Specific features of MDD—for instance, indecisiveness and deflated self-esteem—could also contribute to these patients’ particular use of certain maladaptive coping strategies, such as avoidance [75].

Our findings of depressed patients scoring lower on emotion- and problem-focused coping subscale scores when compared to HCs, on the other hand, contrast some results found in the literature. A number of studies found that depressed patients tended to score higher on the emotion-focused coping subscale [84, 85], while other studies found no significant difference in problem-focused coping subscale scores between depressed patients and HCs [86]. Furthermore, emotion-focused coping was found to be the primary coping style for mildly and moderately depressed patients, while the use of problem-focused coping strategies was not common across depressed patients and healthy controls alike [87]. These results were despite an established inverse relationship between problem-focused coping and MDD [45, 79, 85], but are also partially supported by Folkman and Lazarus (1986), who believe that while problem-focused coping would be effective in the long term, emotion-focused coping would be more effective in the short term. One possible explanation could be that depressed patients employ emotion-focused coping strategies to avoid experiencing high amounts of stress (resulting from directly confronting stressful situations and/or thinking about practical solutions to solve their problems) in the short term and subsequently become accustomed to employing passive approaches when faced with adversity [86, 88]. In our sample, there was a greater proportion of patients with moderate–severe depression. Thus, it is understandable that their predominant mode of coping is dysfunctional, while the utilisation of emotion- and problem-based coping might be variable. It is also possible that the depressive symptoms that they experience have an influence over the coping strategies they choose to employ.

We also found that dysfunctional coping strategies, particularly negative thinking (as opposed to positive reframing), denial, and self-blame, were strong predictors of a positive MDD status. These results seem to be consistent with those of previous studies—for example, repetitive negative thinking (RNT), particularly in the form of rumination, has been shown to increase the risk of depression onset, regardless of whether subjects reported depressive symptoms at the time of study recruitment [89]. Numerous longitudinal studies have also found that elevated levels of rumination and/or worry predict future depressive symptoms, making RNT a MDD risk factor [90, 91]. This can possibly be explained by how RNT is generally characterised by self-focused, repetitive, and negative thinking patterns (i.e., ruminating on negative inferences following stressful events, or the causes and consequences of symptoms experienced), which could represent a cognitive vulnerability factor that influences the interpretation of life events [92, 93]. This would therefore confer vulnerability to the development of MDD and other mood disorders [94].

Our finding that the tendency to engage in both denial and self-blame when faced with stressful events is a strong predictor of a positive MDD status also agrees with the existing literature, which demonstrates that the use of such coping strategies could contribute to the development of MDD [95]. Denial is typically used in an attempt to reject the reality of a stressful event, while self-blame, which is often associated with complex emotions such as guilt, shame, and disgust, involves the excessive blaming of oneself for the occurrence of stressful events [96, 97]. Both are passive coping strategies, which have been shown to have debilitating mental health effects if used frequently [98]. The reason for this could be that since the origin of stress has not been dealt with, the negative emotions associated with the stressful event continue to persist, which could then lead to the manifestation of depressive symptoms [99].

The results of our ROC analyses revealed that the dysfunctional coping style, as measured by the Brief COPE questionnaire, can distinguish between depressed patients and healthy controls with excellent accuracy. A threshold value of 24 had optimal sensitivity and specificity values for screening for, and classifying, depressed patients.

Taken together, these findings highlight the need for clinicians to conduct multidimensional assessments of MDD vulnerability that examine the interplay amongst all possible risk factors [93].

### Strengths and limitations

The main strength of our study is that it explores the predictive and diagnostic potential of both personality and coping styles for MDD, along with how well they can differentiate between depressed patients and healthy individuals. While most research on personality and MDD has focused specifically on neuroticism and/or extraversion, our study examines how MDD associates with each of the Big Five personality dimensions [100]. It is also the first of its kind to be conducted in both the Singaporean and wider Asian context. Our findings, while preliminary, provide evidence of how the diagnosis and treatment of MDD can be improved when personality and coping styles are sufficiently considered when diagnosing for MDD, given their potential implications on disease progression and treatment outcomes.

However, our study also presents a number of limitations. First, participants were recruited via convenience sampling, meaning that our findings cannot be generalised to the larger population [101]. Second, there were approximately three times more female than male participants, which could raise concerns surrounding sex as a confounding factor. However, this ratio appears to reflect a reality that is supported by epidemiological data, which found that females are about twice as likely as males to develop MDD during their lifetime [12, 102]. Furthermore, males were repeatedly found to only be half as likely to seek help for mental health concerns from general practitioners or mental health professionals [103]. Third, our sample size was relatively small and comprised subjects from only one centre, which limited the ability to generalise findings of our study. Fourth, our study was cross-sectional, where MDD patients were recruited and assessed at a time when they were either mildly, moderately, or severely depressed. We are therefore unable to establish causal relationships between personality traits and coping styles with MDD status. The main objective of our study was, however, to explore the potential of personality traits and coping styles to be diagnostic markers for MDD, as well as their ability to differentiate patients with MDD from healthy controls.

## Conclusions

In conclusion, the present study provides empirical evidence for the diagnostic and predictive potential of personality and coping styles for MDD in the clinical setting, as they can potentially be used as indicators of an individual’s increased predisposition to MDD. It also demonstrates the ability of both personality and coping styles to differentiate between depressed patients and HCs with notable accuracy. Given the undeniable implications of personality and coping styles on MDD disease progression and treatment response, it is vital that they be included as part of a holistic psychiatric assessment to allow for MDD diagnoses and treatment in the local and wider Asian contexts to be improved. Further studies involving longitudinal follow-ups are also needed, during which a population of healthy individuals are first assessed for their personality and coping styles at baseline. This would allow for any differences post-MDD diagnosis to be examined and, in turn, distinguish between the role of personality and coping styles as predisposing factors to MDD, or as resulting symptoms of MDD.

## Supplementary Information

**Additional file 1.** Supplemental material for this article is available online.

## Data Availability

The datasets used and/or analysed during the current study are available from the corresponding author on reasonable request.

## Abbreviations

MDD: Major depressive disorder
DSM-5: Fifth Edition of the *Diagnostic and Statistical Manual of Mental Disorders*
HC: Healthy control
HAM-D: Hamilton Rating Scale for Depression
TIPI: Ten-Item Personality Inventory
COPE: Coping Orientation to Problems Experienced
CI: Confidence interval
SD: Standard deviation
OR: Odds ratio
ROC: Receiver operating characteristic
PPV: Positive predictive value
NPV: Negative predictive value
PPD: Postpartum depression
RNT: Repetitive negative thinking
