# Supplementary material for: The diagnostic and predictive potential of personality traits and coping styles in major depressive disorder
Source: BMC Psychiatry. 2022 Apr 28;22:301. doi: 10.1186/s12888-022-03942-y (PMC9047339; doi:10.1186/s12888-022-03942-y)
Supplement: Supplementary file 1 — Additional file 1. Supplemental material for this article is available online. [file 12888_2022_3942_MOESM1_ESM.docx]

**Supplementary Material**

**Figure 1. Pearson Correlations Between HAM-D Total Score and Personality (TIPI).**


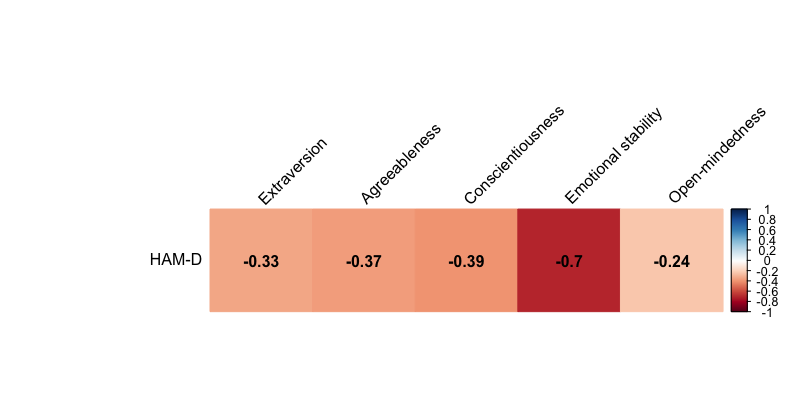


*Note*. Values indicate correlation coefficients and intensity of colours indicate magnitude of these correlation coefficients. Negative correlation coefficients have red backgrounds.

All *p* values < .001.

**Table 1. Results of multiple regression analyses for TIPI personality types predicting HAM-D total score**

| Predictor | Standardised β | *t*-statistic | *p*-value |
| --- | --- | --- | --- |
| Extraversion | - 0.14 | - 2.17 | .032 |
| Agreeableness | - 0.11 | - 1.79 | .076 |
| Conscientiousness | - 0.17 | - 2.66 | .009 |
| Emotional Stability | - 0.57 | - 7.96 | < .001 |
| Open-mindedness | - 0.04 | 0.56 | .574 |

**Figure 2. Pearson Correlations Between HAM-D Categories and Personality (TIPI).**


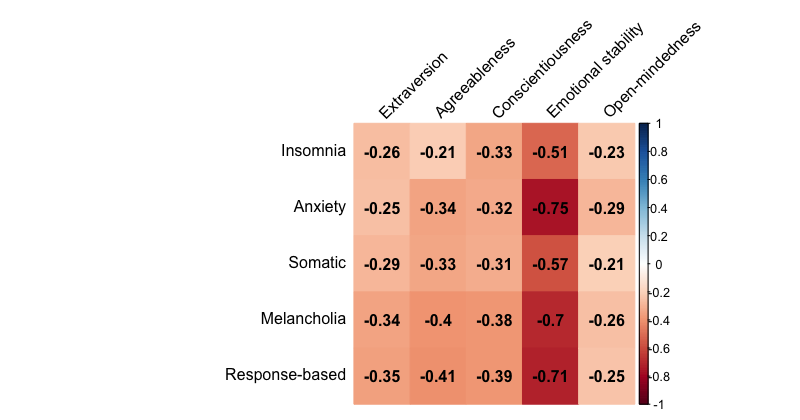


*Note*. Values indicate correlation coefficients and intensity of colours indicate magnitude of these correlation coefficients. Negative correlation coefficients have red backgrounds.

All *p* values ≤ 0.05.

**Figure 3. Pearson Correlations Between HAM-D Total Score and Coping Styles (Brief COPE).**


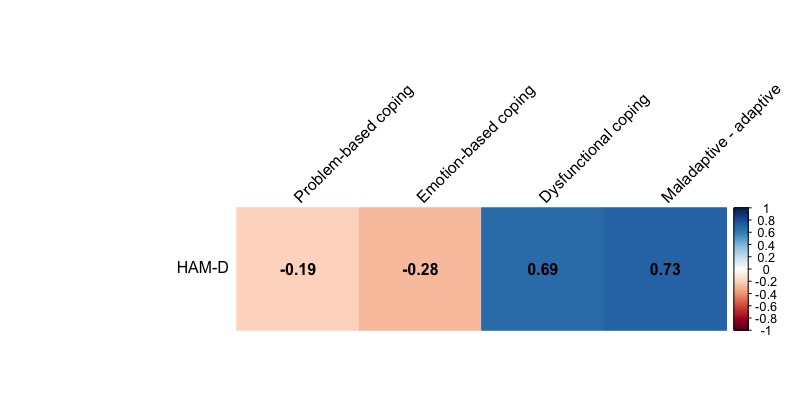


*Note*. Values indicate correlation coefficients and intensity of colours indicate magnitude of these correlation coefficients. Negative correlation coefficients have red backgrounds; positive correlation coefficients have blue backgrounds.

All *p* values ≤ .05.

**Table 2. Results of multiple regression analyses for Brief COPE coping styles predicting HAM-D total score**

| Predictor | Standardised β | *t*-statistic | *p*-value |
| --- | --- | --- | --- |
| Problem-based coping | -0.09 | -1.36 | .176 |
| Emotion-based coping | -0.29 | -4.50 | < .001 |
| Dysfunctional coping | 0.71 | 12.94 | < .001 |

*Note*. HAM-D = Hamilton Depression Rating Scale. Problem-based coping, emotion-based coping and dysfunctional coping are facets obtained from the Brief COPE questionnaire.

**Figure 4. Pearson Correlations Between HAM-D Categories and Coping Styles (Brief COPE).**


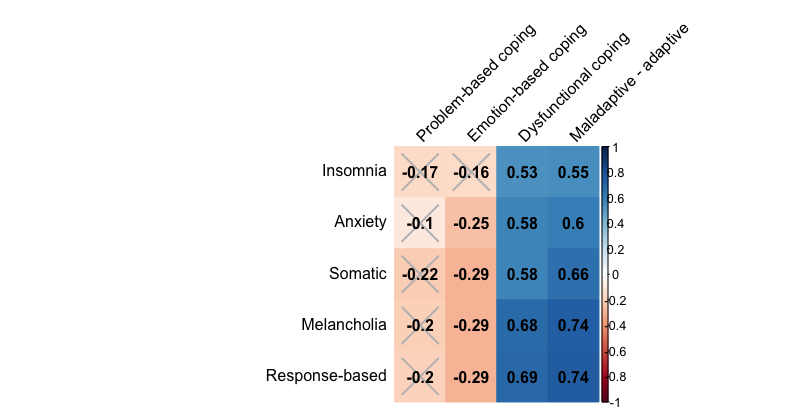


*Note*. Values indicate correlation coefficients and intensity of colours indicate magnitude of these correlation coefficients. Negative correlation coefficients have red backgrounds; positive correlation coefficients have blue backgrounds.

Non-significant findings (*p* > .05) are crossed out. All other *p* values ≤ .05.

**Table 3. Multivariate Multiple Regression of HAM-D Categories on Personality (TIPI)**

|  | Insomnia | | | Anxiety | | | Somatic | | | Melancholia | | | Response-based | | |
| --- | --- | --- | --- | --- | --- | --- | --- | --- | --- | --- | --- | --- | --- | --- | --- |
|  | Standardised β | *t*-statistic | *p*-value | Standardised β | *t*-statistic | *p*-value | Standardised β | *t*-statistic | *p*-value | Standardised β | *t*-statistic | *p*-value | Standardised β | *t*-statistic | *p*-value |
| Extraversion | -0.10 | -1.21 | .228 | 0.00 | 0.02 | .984 | -0.13 | -1.70 | .091 | -0.15 | -2.26 | .026 | -0.16 | -2.51 | .013 |
| Agreeableness | -0.02 | -0.24 | .811 | 0.00 | -0.96 | .337 | -0.13 | -1.76 | .080 | -0.16 | -2.46 | .015 | -0.16 | -2.59 | .011 |
| Conscientiousness | -0.17 | -2.20 | .029 | -0.06 | -1.10 | .273 | -0.12 | -1.68 | .096 | -0.15 | -2.39 | .018 | -0.15 | -2.53 | .013 |
| Emotional Stability | -0.40 | -4.55 | < .001 | -0.69 | -9.99 | < .001 | -0.45 | -5.34 | < .001 | -0.54 | -7.65 | < .001 | -0.55 | -7.98 | < .001 |
| Open-mindedness | -0.03 | -0.39 | .698 | -0.06 | -0.84 | .405 | -0.02 | 0.30 | .764 | 0.01 | 0.21 | .831 | 0.03 | 0.52 | .607 |

**Table 4. Multivariate Multiple Regression of HAM-D Categories on Coping Styles**

|  | Insomnia | | | Anxiety | | | Somatic | | | Melancholia | | | Response-based | | |
| --- | --- | --- | --- | --- | --- | --- | --- | --- | --- | --- | --- | --- | --- | --- | --- |
|  | Standardised β | *t*-statistic | *p*-value | Standardised β | *t*-statistic | *p*-value | Standardised β | *t*-statistic | *p*-value | Standardised β | *t*-statistic | *p*-value | Standardised β | *t*-statistic | *p*-value |
| Problem-based coping | -0.14 | -1.70 | .091 | 0.03 | 0.39 | .695 | -0.11 | -1.45 | .149 | -0.09 | -1.44 | .151 | -0.09 | -1.42 | .159 |
| Emotion-based coping | -0.13 | -1.58 | .118 | -0.32 | -4.18 | < .001 | -0.29 | -3.87 | < .001 | -0.30 | -4.75 | < .001 | -0.30 | -4.81 | < .001 |
| Dysfunctional coping | 0.55 | 7.92 | < .001 | 0.60 | 9.25 | < .001 | 0.61 | 9.56 | < .001 | 0.71 | 12.98 | < .001 | 0.72 | 13.29 | < .001 |
